# The Association of Thyroid Nodules with Metabolic Status: A Cross-Sectional SPECT-China Study

**DOI:** 10.1155/2018/6853617

**Published:** 2018-03-06

**Authors:** Yi Chen, Chunfang Zhu, Yingchao Chen, Ningjian Wang, Qin Li, Bing Han, Li Zhao, Chi Chen, Hualing Zhai, Yingli Lu

**Affiliations:** Institute and Department of Endocrinology and Metabolism, Shanghai Ninth People's Hospital, Shanghai Jiao Tong University School of Medicine, Shanghai, China

## Abstract

**Purpose:**

The aim of this study was to investigate the association of thyroid nodules (TNs) and their ultrasound (US) characteristics related to malignancy with metabolic status.

**Methods:**

The data were obtained from a cross-sectional study (SPECT-China, 2014-2015). The study included 9898 participants older than 18 years. Participants underwent several checkups, which included the measurement of anthropometric parameters, blood pressure, TSH levels, glucose, and lipid profiles. TN and nonalcoholic fatty liver disease (NAFLD) were diagnosed by US. TN US characteristics, including microcalcification and a taller-than-wide shape, were recorded.

**Results:**

Participants with TN [TN(+)] had a higher prevalence of metabolic syndrome (Met-S), obesity, central obesity, hyperlipidaemia, diabetes, hypertension, and NAFLD, especially women (all *P* ≤ 0.001). After full adjustment, logistic regression analysis indicated that metabolic syndrome, obesity, central obesity, and hyperlipidaemia were all independent risk factors for the increased prevalence of TN in both genders (*P* < 0.05). In terms of TN US imaging characteristics associated with malignancy, being female with obesity, central obesity, and NAFLD had 1.91-fold, 2.09-fold, and 1.75-fold increased risks of developing a taller-than-wide nodule (*P* = 0.014, 0.004, and 0.027, resp.).

**Conclusions:**

The status of metabolic disorders might be associated with higher risks of TN in both genders. In women, obesity, central obesity, and NAFLD might contribute to the development of a taller-than-wide nodule. The potential role of metabolic status in the pathogenesis of the thyroid nodule and thyroid cancer remains to be elucidated.

## 1. Introduction

The American Thyroid Association Guidelines Task Force on Thyroid Nodules and Differentiated Thyroid Cancer [1] recently declared that thyroid nodules (TNs) are a common clinical problem, and differentiated thyroid cancer is becoming increasingly prevalent. Currently, high-resolution ultrasound (US) can detect TN in 19%–68% of randomly selected individuals in a population [2, 3]. The clinical importance of thyroid nodules is the need to exclude thyroid cancer, which occurs in 7%–15% of a population [4]. In China, the prevalence of thyroid nodules is high, even in healthy adults, at approximately 30 to 50% [5–7], and thyroid cancer has become one of the ten most common cancers in the Chinese population, especially among women [8]. Similarly, one study predicted that papillary thyroid carcinoma will become the third most common cancer in women in the United States by 2019 [9]. Based on these issues, further study of the relevant risk factors for TN and thyroid cancer is required.

The American Association of Clinical Endocrinologists (AACE)/Associazione Medici Endocrinologi (AME)/European Thyroid Association (ETA) [10] declared that TNs are more common in elderly people, women, people with iodine deficiency, and people with a history of radiation exposure. Some thyroid function factors (i.e., TSH and thyroid antibodies) might contribute to the growth and progression of TN [11], and TNs are also closely related to a greater waist circumference (WC), higher triglyceride (TG) level, HOMA-IR, and HbA1c [12, 13]. Among the risk factors for thyroid cancer, in addition to age, sex, radiation exposure history, and family history, other factors [4, 14, 15] including obesity and central obesity may contribute to its aetiology.

Currently, the association of metabolic status with TN and thyroid cancer has not been fully investigated. The metabolic mechanisms facilitating the development of TN and thyroid cancer are complex and have not been confirmed. The data used in this study were from a large investigation, the Survey on Prevalence in East China for Metabolic Diseases and Risk Factors (SPECT-China), which was performed in 2014-2015. The objective of the present study was to investigate whether different metabolic risk factors and metabolic diseases, including obesity, central obesity, hyperlipidaemia, hypertension, diabetes, metabolic syndrome (Met-S), and nonalcoholic fatty liver disease (NAFLD), were associated with TN and their US imaging characteristics related to malignancy.

## 2. Materials and Methods

### 2.1. Study Participants

SPECT-China [16, 17] is a population-based survey that assessed the prevalence of metabolic diseases and risk factors in East China. A stratified and cluster sampling method was used. From February 2014 to December 2015, this study was performed in Shanghai, Zhejiang, Jiangxi, Jiangsu, and Anhui and in 22 sites in East China. Adults aged 18 years or older who were Chinese citizens and had lived in their current residence for more than 6 months were invited to participate in our study. Those with severe communication problems, with acute illness, or who were unwilling to participate were excluded. All participants provided written informed consent before data collection. The study protocol was approved by the ethics committee of the Shanghai Ninth People's Hospital, Shanghai Jiao Tong University School of Medicine. All procedures used were in accordance with the ethical standards of the responsible committee on human experimentation (institutional and national) and with the Helsinki Declaration of 1975, as revised in 2008 [18, 19].

**Figure 1:**
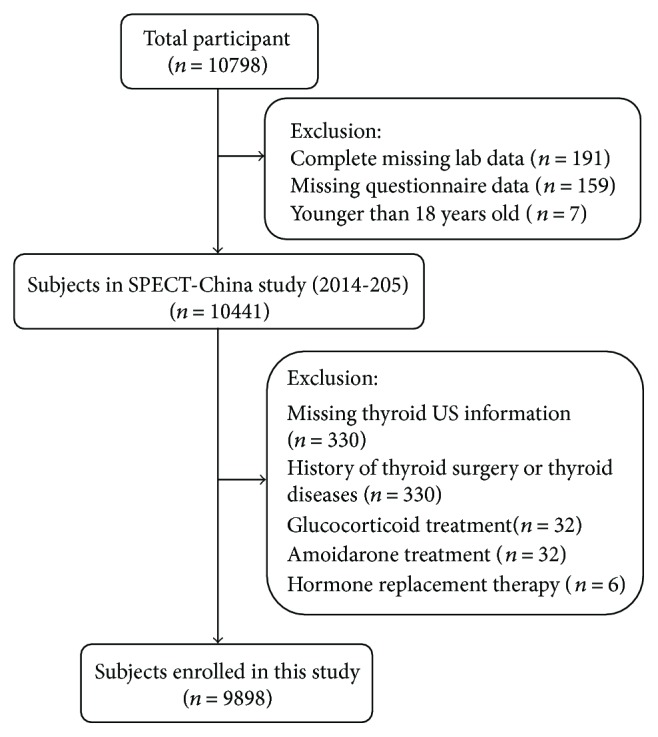
Flowchart of participants' inclusion and exclusion.

Our study initially enrolled 10,441 participants older than 18 years [17–19]. Participants missing thyroid ultrasound (US) information (*n* = 330) and those with a history that included thyroid surgery, thyroid diseases (including hyperthyroidism, hypothyroidism, subacute thyroiditis, and radioactive iodine treatment history) (*n* = 174), glucocorticoid treatment (*n* = 32), amiodarone treatment (*n* = 1), or hormone replacement therapy including androgen and oestrogen (*n* = 6) were excluded. Finally, 9898 subjects were included in the final analysis.  Figure 1 shows the inclusion and exclusion of participants in this analysis.

When we analysed the association between TN and NAFLD, participants missing liver ultrasonographic results (*n* = 213) and those with a history of excessive consumption of alcohol (male > 20 g/d, female > 10 g/d) [17] (*n* = 598), schistosomal hepatic disease (*n* = 5), or self-reported viral hepatitis (including hepatitis B and hepatitis C viruses) (*n* = 110) were excluded; finally, 8977 subjects were included in this analysis.

### 2.2. Data Collection

All data collection in this study was performed by the same staff from the Department of Endocrinology and Metabolism at the Shanghai Ninth People's Hospital, Shanghai Jiao Tong University School of Medicine. All staffs successfully completed a standard training programme that familiarized them with the specific tools and methods used. A standard questionnaire was administered by trained staff to obtain information on demographic characteristics, personal and family medical history, and risk factors in their daily lives. Weight, height, and waist and hip circumferences were measured according to a standard protocol. Blood pressure (BP) was measured on the nondominant arm 3 times consecutively with a 1-minute interval between the measurements while the participant was in a seated position after 5 minutes of rest [20]. All anthropometric measurements were conducted at the same time as the serum sample collection.

### 2.3. Laboratorial Assays

Serum samples for laboratorial assays were obtained by venipuncture after an 8-hour fast from 0700 to 1000 h in the morning. Blood samples were stored at −20°C when collected and shipped by air in dry ice to one central laboratory, which was certified by the College of American Pathologists (CAP), within 2–4 hours of collection.

Fasting plasma glucose (FPG), low-density lipoprotein (LDL), high-density lipoprotein (HDL), triglycerides (TG), and total cholesterol (TC) were measured by Beckman Coulter AU 680 (Brea, USA). Insulin was detected by chemiluminescence method (Abbott i2000 SR, Chicago, USA). Glycated hemoglobin (HbA1c) was assessed by high-performance liquid chromatography (MQ-2000PT, Medconn, Shanghai, China). Thyroid peroxidase antibody (TPOAb), thyroglobulin antibody (TgAb), thyroid stimulating hormone (TSH), triiodothyronine (T_3_), and thyroxine (T_4_) were measured by the chemiluminescence immunoassay (Siemens, immulite 2000, Erlangen, Germany).

### 2.4. Thyroid and Liver Ultrasonography

Thyroid and liver US examinations were performed by the same registered physicians who were unaware of the biochemical and histological data of the participants and had professional certificates for ultrasonography awarded by the Ministry of Health of China for the use of B-mode US imaging (M7, Mindray ShenZhen, P.R. China). The characteristics of the thyroid parenchyma were described according to the echogenicity and homogeneity. Nodule characteristics, including microcalcification and taller-than-wide shape, were recorded. Hepatic steatosis was defined as a diffuse increase of the fine echoes in the liver parenchyma compared to that in the kidney or spleen parenchyma based on standard criteria [21].

### 2.5. Definition of Variables

Thyroid nodule: ≥2 mm in diameter. Microcalcification: calcification < 2 mm.

Obesity was defined based upon BMI measures ≥ 28 kg/m^2^. Central obesity was defined as a waist circumference ≥ 80 cm in women and ≥90 cm in men [20]. Based on the American Diabetes Association 2014 criteria, diabetes was defined as a previous diagnosis by healthcare professionals, fasting plasma glucose ≥ 7.0 mmol/L, or HbA1c ≥ 6.5%. Hyperlipidaemia was defined as total cholesterol ≥ 6.22 mmol/L, triglycerides ≥ 2.26 mmol/L, LDL-C ≥ 4.14 mmol/L, HDL-C < 1.04 mmol/L, or a self-reported previous diagnosis of hyperlipidaemia by physicians. Hypertension was defined as a systolic blood pressure of 140 mm Hg or higher, a diastolic blood pressure of 90 mm Hg or higher, or current use of antihypertensive treatment. Metabolic syndrome (Met-S) was defined based on the International Diabetes Federation Criteria (2005). A person with MS must have abdominal obesity (waist circumference: male ≥ 90 cm, female ≥ 80 cm, or BMI ≥ 30 kg/m^2^) and any two of the following four parameters: (1) elevated TG ≥ 1.7 mmol/L or treatment for dyslipidaemia; (2) reduced HDL < 1.03 mmol/L in men or <1.29 mmol/L in women or treatment for dyslipidaemia; (3) elevated blood pressure (systolic blood pressure ≥ 130 or diastolic blood pressure ≥ 85 mmHg) or treatment of hypertension; and (4) elevated fasting plasma glucose ≥ 5.6 mmol/L or a history of type 2 diabetes [22].

### 2.6. Statistical Analysis

We performed analyses of the survey using IBM SPSS Statistics, Version 22 (IBM Corporation, Armonk, NY, USA). All analyses were two-sided. A *P* value < 0.05 indicated significance. Continuous variables are presented as the mean (±standard deviation) values, and categorical variables are presented as percentages. The prevalence of TN was calculated among all participants. The prevalence of nodule characteristics, including microcalcification and a taller-than-wide shape, was calculated among the participants with thyroid nodules. Continuous variables were compared using Student's *t*-test. The Mann–Whitney *U* test was used for nonnormally distributed continuous variables, and Pearson's *χ*^2^ test was used for dichotomous variables. The associations of thyroid nodules and their US imaging characteristics with metabolic diseases (categorical variables) were assessed by logistic regression. The regression models were adjusted for age, smoking history (including current and past), TSH, and waist-to-hip ratio (but not including obesity, central obesity, and metabolic syndrome.). The results are expressed as odds ratios (95% confidence interval).

Body mass index (BMI) was calculated as weight in kilograms divided by height in metre squared. Insulin resistance was estimated by the homeostatic model assessment (HOMA-IR) index: [fasting insulin(mIU/L)] × [FPG (mmol/L)]/22.5.

## 3. Results

### 3.1. Clinical Characteristics of Participants with and without TN

A total of 9898 subjects (4117 men; 5781 women) were enrolled in this study. The mean age was 53.34 ± 13.07 years, and the mean BMI was 24.52 ± 3.52 kg/m^2^. The prevalence of TN was 50.2% (41.3% in men and 56.5% in women). Compared to those without thyroid nodules [TN(−)], men with thyroid nodule(s) [TN(+)] were significantly older; had a significantly greater BMI, waist circumference, waist-to-hip ratio, and systolic pressure; had higher levels of FPG, HOMA-IR, HbA1c, LDL, TG, T_4_, TPOAb, and TgAb; and had lower levels of HDL (*P* < 0.05). For women, significantly higher levels of TC and diastolic pressure were found as well. Furthermore, participants with TN, for both genders, had a higher prevalence of metabolic syndrome, obesity, central obesity, hyperlipidaemia, diabetes, hypertension, and NAFLD (all *P* ≤ 0.001). The characteristics of the study subjects with and without TN are summarized in Table 1.

### 3.2. Prevalence of TN according to the Presence of Metabolic Diseases

The participants were classified into different subgroups according to whether they presented with metabolic diseases (Table 2). The prevalence of TN was significantly increased in subjects with metabolic syndrome, obesity, central obesity, hyperlipidaemia, diabetes, hypertension, and NAFLD for both genders (all *P* ≤ 0.001).

### 3.3. The Relationship between TN and Metabolic Diseases

**Figure 2:**
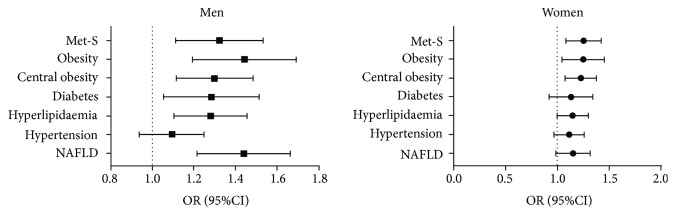
Associations of TN with metabolic diseases. The associations were analysed using logistic regression analysis. The regression models were adjusted for age, smoking history (including current and past), TSH, and waist-to-hip ratio (but not including obesity, central obesity, and metabolic syndrome).

Then, the adjusted odds ratios (ORs) for TN in both genders were calculated. After adjusting for age, smoking history (including current and past), TSH, and waist-to-hip ratio (but not including obesity, central obesity, and metabolic syndrome), the logistic regression analysis indicated that metabolic syndrome (men: OR = 1.31, 95% CI 1.12, 1.54, *P* = 0.001; women: OR = 1.24, 95% CI 1.09, 1.43, *P* = 0.002), obesity (men: OR = 1.43, 95% CI 1.20, 1.70, *P* < 0.001; women: OR = 1.24, 95% CI 1.05, 146, *P* = 0.013), central obesity (men: OR = 1.29, 95% CI 1.12, 1.49, *P* < 0.001; women: OR = 1.22, 95% CI 1.08, 1.38, *P* = 0.001), and hyperlipidaemia (men: OR = 1.27, 95% CI 1.11, 1.46, *P* = 0.001; women: OR = 1.14, 95% CI 1.00, 1.30, *P* = 0.045) were associated with TN, whereas diabetes and NAFLD were only associated with TN in men (OR = 1.27, 95% CI 1.06, 1.52, *P* = 0.010 and OR = 1.43, 95% CI 1.22, 1.67, *P* < 0.001, resp.). Hypertension was not an independent significant risk factor for the increased prevalence of TN in both genders (*P* > 0.05) (Figure 2).

### 3.4. The Association of the US Imaging Characteristics of TN and Metabolic Diseases

Among the participants with TN (*n* = 4965), the prevalence of TN with microcalcification was 1.1% (1.3% in men and 1.0% in women), and the prevalence of taller-than-wide-shaped TN was 2.3% (2.0% in men and 2.5% in women).

We evaluated the relationship between the above US characteristics associated with malignancy (microcalcification and a taller-than-wide shape) with metabolic diseases (including metabolic syndrome, obesity, central obesity, hyperlipidaemia, diabetes, hypertension, and NAFLD). Among women with TN, the prevalence of taller-than-wide TN was significantly increased in participants with obesity, central obesity, and NAFLD (all *P* < 0.01). No significant difference was found among men with TN (Table 2).

Then, we calculated the adjusted ORs for these US characteristics associated with malignancy and found that women with obesity, central obesity, and hypertension had 1.91-fold (95% CI 1.14, 3.21, *P* = 0.014), 2.09-fold (95% CI 1.27, 3.46, *P* = 0.004), and 1.75-fold (95% CI 1.07, 2.87, *P* = 0.027) increased risks of developing a taller-than-wide nodule. For men, metabolic syndrome had a marginal correlation with higher risks of a nodule with microcalcification (OR = 2.67, 95% CI 1.01, 7.04, *P* = 0.048). No other significant association was detected.

## 4. Discussion

Thyroid US is strongly recommended as the first-line screening diagnostic test to detect thyroid lesions [23] due to its safety, noninvasive nature, and high sensitivity, and it has also been widely used to stratify the risk of malignancy in thyroid nodules and aid decision-making about whether fine needle aspiration is indicated. Researchers have consistently reported that several US grey-scale features in multivariate analyses were found to be highly likely to be malignant, including the presence of nodules with microcalcification and a taller-than-wide shape measured on a transverse view [1, 10]. In this study, the association of TN and their US characteristics with different metabolic diseases including obesity, central obesity, hyperlipidaemia, hypertension, diabetes, Met-S, and NAFLD was explored.

A previous study [24–27] reported that TNs are closely related to several components of Met-S. In this study, regardless of age, participants with TN had a significantly greater BMI, waist circumference, waist-to-hip ratio, and blood pressure as well as worse glucose and lipid profiles for both genders. Furthermore, a higher prevalence of metabolic syndrome, obesity, central obesity, hyperlipidaemia, diabetes, and hypertension was seen in the TN(+) group as well. Given the findings that the subjects in the TN(+) group had worse metabolic parameters with a higher prevalence of metabolic diseases, we evaluated the adjusted ORs for TN. After full adjustment, participants of both genders with metabolic syndrome, obesity, central obesity, and hyperlipidaemia had significantly greater risks of TN, while diabetes was associated with TN in only men. To our knowledge, the literature about the relationship between NAFLD and TN remains scarce. Most recently, one published study [26] reported that the prevalence of TN in NAFLD was significantly increased in women; however, after logistic regression analyses, there was no significant difference observed. In this study, after full adjustment, the risk of TN increased by 43% in men with NAFLD. Although higher blood pressure was found in subjects with TN and the prevalence of TN was also significantly higher in participants with hypertension, there was no association between TN and hypertension after full adjustment.

In terms of thyroid cancer, currently, most studies concerning thyroid cancer and metabolic factors have focused on obesity. Several researchers have indicated that overweight and obesity are related to a modestly increased thyroid cancer risk for both genders [28–30], while similar results were found in only women in other studies [31–33]. Consistent with a previous study, in this study, the prevalence of taller-than-wide TN was significantly increased in participants with obesity and central obesity in only women. According to the logistic regression analysis, the adjusted ORs of taller-than-wide nodules were 1.91 and 2.09 in women with obesity and central obesity, respectively. Yeo et al. found that compared with nondiabetic subjects, women with preexisting DM have an increased risk of thyroid cancer [34]. Similarly, another study that focused on diabetes and thyroid cancer risk also indicated that diabetes increases the risk of differentiated thyroid cancer [35]. However, another study found the opposite result, showing that there was an inverse association between glucose and thyroid cancer risk in women [31]. In this present study, among women with TN, the prevalence of nodules with microcalcifications or a taller-than-wide shape had no significant association with diabetes. Balkan et al. [36] reported that Met-S was not a significant risk factor of thyroid cancer following logistic regression analysis. A similar result was found in which there was no association between some metabolic factors (blood pressure, cholesterol, triglycerides, and a combined Met-S score) and thyroid cancer [31]. This study showed a 2.67-fold increase in the risk for TN with microcalcifications among men with Met-S after full adjustment. Furthermore, women with NAFLD had a 1.75-fold increased risk of developing taller-than-wide nodules. Thus, data from this study suggested a potential role of metabolic factors besides obesity on the pathogenesis of thyroid cancer, which expanded our current understanding of the relationship between metabolism status and thyroid cancer.

Our study has several important strengths. First, we evaluated a relatively large sample of participants to examine the association of TN and their US findings related to malignancy with almost all common metabolic diseases. To our knowledge, this is the most comprehensive report to date addressing this issue. Second, all anthropometric measurements and questionnaires were completed by the same trained research group using strong quality controls. Third, we avoided a clinic-based population, and instead, community-dwelling participants living in multiple sites in China were recruited so that their results may be more representative.

However, our study also has several limitations. First, owing to the cross-sectional study nature, no causal inference can be drawn, and the reverse effect of TN and thyroid cancer causing metabolic status changes still needs to be excluded. Second, the gold standard for diagnosing a thyroid nodule, especially for thyroid cancer and NAFLD, is biopsy, and the use of ultrasonography has limitations. However, thyroid and liver biopsies may not be feasible in such a large sample. In our study, all liver and thyroid ultrasounds were performed by the same ultrasound physicians, minimizing the interobserver variability. Third, additional nodule characteristics associated with malignancy, such as hypoechoic nodules, nodules with irregular and indefinite borders, were not recorded, which needs to be further explored. Finally, self-reporting of viral hepatitis may have introduced recall bias.

## 5. Conclusion

The status of metabolic disorders might be associated with higher risks of TN in both genders. In women, obesity, central obesity, and NAFLD might contribute to the development of a taller-than-wide nodule. The potential role of metabolic status in the pathogenesis of thyroid nodules and thyroid cancer remains to be elucidated.

## Figures and Tables

**Table 1:** General characteristics of all subjects with and without thyroid nodules.

	Men			Women		
	TN(−)	TN(+)	*P*	TN(−)	TN(+)	*P*
*N* (%)	2417 (58.7)	1700 (41.3)	—	2516 (43.5)	3265 (56.5)	—
Age (year)	51.86 ± 13.26	57.25 ± 12.36	<0.001	48.41 ± 12.36	56.19 ± 12.39	<0.001
Smokers (%)	53.3	55.6	0.154	2.6	2.9	0.537
*Metabolic factors*						
BMI (kg/m^2^)	24.64 ± 3.39	25.19 ± 3.23	<0.001	23.74 ± 3.65	24.70 ± 3.56	<0.001
WC (cm)	83.43 ± 9.53	85.65 ± 9.59	<0.001	75.80 ± 9.65	79.45 ± 9.95	<0.001
WHR	0.89 ± 0.07	0.90 ± 0.08	<0.001	0.82 ± 0.07	0.85 ± 0.11	<0.001
FPG (mmol/L)	5.62 ± 1.38	5.82 ± 1.68	0.001	5.43 ± 1.15	5.68 ± 1.48	<0.001
HOMA-IR	1.46 ± 2.94	1.54 ± 2.57	0.011	1.45 ± 1.82	1.65 ± 1.99	<0.001
HbA1c (%)	5.55 ± 0.94	5.73 ± 1.11	<0.001	5.33 ± 0.83	5.57 ± 0.95	<0.001
LDL (mmol/L)	3.05 ± 0.76	3.12 ± 0.75	0.001	2.99 ± 0.79	3.15 ± 0.81	<0.001
HDL (mmol/L)	1.37 ± 0.32	1.33 ± 0.32	<0.001	1.49 ± 0.30	1.46 ± 0.32	0.007
TG (mmol/L)	1.88 ± 2.01	1.89 ± 1.68	0.023	1.40 ± 0.96	1.59 ± 1.36	<0.001
TC (mmol/L)	5.14 ± 1.12	5.17 ± 1.11	0.331	5.04 ± 1.01	5.25 ± 1.21	<0.001
SBP (mmHg)	133.40 ± 20.69	136.22 ± 20.89	<0.001	126.28 ± 21.66	134.02 ± 22.35	<0.001
DBP (mmHg)	82.02 ± 12.99	82.44 ± 13.04	0.310	76.41 ± 12.73	78.45 ± 12.99	<0.001
*Metabolic diseases*						
Met-S (%)	18.3	24.1	<0.001	20.2	32.5	<0.001
Obesity (%)	15.2	19.1	0.001	11.1	16.5	<0.001
Central obesity (%)	26.4	33.4	<0.001	34.1	48.7	<0.001
Diabetes (%)	12.9	18.6	<0.001	8.5	14.9	<0.001
Hyperlipidaemia (%)	40.2	47.2	<0.001	26.5	37.5	<0.001
Hypertension (%)	47.8	57.6	<0.001	32.8	49.1	<0.001
NAFLD (%)	29.7	35.7	<0.001	19.1	25.8	<0.001
*Thyroid hormones*						
TSH (mIU/L)	2.44 ± 2.68	2.47 ± 3.76	0.586	3.12 ± 4.77	3.01 ± 2.80	0.748
T_3_ (nmol/L)	1.80 ± 0.56	1.79 ± 0.50	0.581	1.72 ± 0.39	1.76 ± 0.41	<0.001
T_4_ (nmol/L)	112.80 ± 22.42	115.19 ± 23.81	0.001	113.63 ± 20.59	116.33 ± 20.55	<0.001
TPOAb (U/ml)	66.26 ± 198.51	76.14 ± 217.03	<0.001	139.92 ± 339.82	139.42 ± 333.46	0.177
TgAb (U/ml)	26.68 ± 56.33	30.77 ± 71.78	0.001	54.41 ± 111.91	54.55 ± 130.44	0.488

BMI: body mass index; WC: waist circumference; WHR: waist to hip ratio; FPG: fasting blood glucose; HOMA-IR: homeostasis model assessment of insulin resistance; HbA1c: glycated hemoglobin; LDL: low-density lipoprotein; HDL: high-density lipoprotein; TG: triglycerides; TC: total cholesterol; SBP: systolic blood pressure; DBP: diastolic blood pressure; Met-S: metabolic syndrome; NAFLD: nonalcoholic fatty liver disease; TSH: thyroid stimulating hormone; T_3_: triiodothyronine; T_4_: thyroxine; TPOAb: thyroid peroxidase antibody; TgAb: thyroglobulin antibody.

**Table 2:** Prevalence of TN and US characteristics (%) according to the status of metabolic diseases.

	Men			Women		
	TN	Microcalcification	Taller-than-wide shape	TN	Microcalcification	Taller-than-wide shape
Met-S	48.2	2.3	1.5	67.6	1.0	3.3
Without Met-S	39.6	1.0	1.9	52.3	0.9	2.1
*P* value	<0.001	0.073	0.596	<0.001	0.731	0.102
Obesity	46.9	1.1	2.8	66.0	0.8	4.1
Without obesity	40.2	1.4	1.8	55.0	1.0	2.2
*P* value	0.001	0.646	0.222	<0.001	0.696	0.007
Central obesity	47.1	1.6	2.5	65.0	1.2	3.4
Without central obesity	39.0	1.2	1.6	50.2	0.7	1.6
*P* value	<0.001	0.498	0.207	<0.001	0.210	0.003
Diabetes	50.3	0.8	1.6	69.5	1.5	3.9
Without diabetes	39.7	1.5	2.1	54.7	0.9	2.2
*P* value	<0.001	0.371	0.550	<0.001	0.318	0.086
Hyperlipidaemia	45.3	1.7	2.5	64.7	1.2	2.5
Without hyperlipidaemia	38.3	1.0	1.6	52.5	0.9	2.5
*P* value	<0.001	0.276	0.168	<0.001	0.361	0.733
Hypertension	45.8	1.3	1.9	66.1	1.3	2.6
Without hypertension	36.3	1.4	2.3	49.6	0.8	2.4
*P* value	<0.001	0.806	0.576	<0.001	0.168	0.572
NAFLD	44.8	1.3	2.0	63.5	1.5	3.8
Without NAFLD	38.3	1.0	2.3	54.0	0.8	2.0
*P* value	<0.001	0.650	0.699	<0.001	0.084	0.008

Met-S: metabolic syndrome; NAFLD: nonalcoholic fatty liver disease.
